# Using ChatGPT to Improve the Presentation of Plain Language Summaries of Cochrane Systematic Reviews About Oncology Interventions: Cross-Sectional Study

**DOI:** 10.2196/63347

**Published:** 2025-03-19

**Authors:** Jelena Šuto Pavičić, Ana Marušić, Ivan Buljan

**Affiliations:** 1 Department of Oncology and Radiotherapy, University Hospital of Split, Spinciceva 1, Split, 21000, Croatia, 385 2155817; 2 Department of Research in Biomedicine in Health, Centre for Evidence-based Medicine, University of Split School of Medicine, Split, Croatia; 3 Department of Psychology, Faculty of Humanities and Social Sciences, University of Split, Split, Croatia

**Keywords:** health literacy, patient education, health communication, ChatGPT, neoplasms, Cochrane, oncology, plain language, medical information, decision-making, large language model, artificial intelligence, AI

## Abstract

**Background:**

Plain language summaries (PLSs) of Cochrane systematic reviews are a simple format for presenting medical information to the lay public. This is particularly important in oncology, where patients have a more active role in decision-making. However, current PLS formats often exceed the readability requirements for the general population. There is still a lack of cost-effective and more automated solutions to this problem.

**Objective:**

This study assessed whether a large language model (eg, ChatGPT) can improve the readability and linguistic characteristics of Cochrane PLSs about oncology interventions, without changing evidence synthesis conclusions.

**Methods:**

The dataset included 275 scientific abstracts and corresponding PLSs of Cochrane systematic reviews about oncology interventions. ChatGPT-4 was tasked to make each scientific abstract into a PLS using 3 prompts as follows: (1) rewrite this scientific abstract into a PLS to achieve a Simple Measure of Gobbledygook (SMOG) index of 6, (2) rewrite the PLS from prompt 1 so it is more emotional, and (3) rewrite this scientific abstract so it is easier to read and more appropriate for the lay audience. ChatGPT-generated PLSs were analyzed for word count, level of readability (SMOG index), and linguistic characteristics using Linguistic Inquiry and Word Count (LIWC) software and compared with the original PLSs. Two independent assessors reviewed the conclusiveness categories of ChatGPT-generated PLSs and compared them with original abstracts to evaluate consistency. The conclusion of each abstract about the efficacy and safety of the intervention was categorized as conclusive (positive/negative/equal), inconclusive, or unclear. Group comparisons were conducted using the Friedman nonparametric test.

**Results:**

ChatGPT-generated PLSs using the first prompt (SMOG index 6) were the shortest and easiest to read, with a median SMOG score of 8.2 (95% CI 8‐8.4), compared with the original PLSs (median SMOG score 13.1, 95% CI 12.9‐13.4). These PLSs had a median word count of 240 (95% CI 232‐248) compared with the original PLSs’ median word count of 364 (95% CI 339‐388). The second prompt (emotional tone) generated PLSs with a median SMOG score of 11.4 (95% CI 11.1‐12), again lower than the original PLSs. PLSs produced with the third prompt (write simpler and easier) had a median SMOG score of 8.7 (95% CI 8.4‐8.8). ChatGPT-generated PLSs across all prompts demonstrated reduced analytical tone and increased authenticity, clout, and emotional tone compared with the original PLSs. Importantly, the conclusiveness categorization of the original abstracts was unchanged in the ChatGPT-generated PLSs.

**Conclusions:**

ChatGPT can be a valuable tool in simplifying PLSs as medically related formats for lay audiences. More research is needed, including oversight mechanisms to ensure that the information is accurate, reliable, and culturally relevant for different audiences.

## Introduction

The significance of health literacy has been well established through numerous studies [1,2], demonstrating its importance not only for individual health care [3] but also within the public health system [4,5]. Various organizations, including the Center for Disease Control and Prevention and the National Institute of Health, have underscored the importance of using plain language in health communication to enhance understanding of medical conditions and patients’ engagement [6,7]. Health literacy is particularly important in oncology, where the advancement in cancer treatment beyond traditional methods has positioned patients and their families even more in the center of making care decisions [8]. Upon receiving a cancer diagnosis, patients often turn to various sources for more information, such as the internet, forums, social support groups, and literature [9,10]. Enhancing patients’ understanding of their conditions has been shown to positively impact patient adherence and clinical outcomes in various chronic disease models by influencing patient behavior [11-13], whereas patients’ struggle to understand complex medical information can adversely affect their adherence to medical advice [14]. However, nearly half of cancer patients struggle to understand the information about their treatment options from scientific literature [15]. Even though the official recommendation of the American Medical Association is that health information should be written at the reading level of the sixth grade in the US education system [16], the complexity of web-based cancer information often exceeds the reading and comprehension abilities of an average person, failing to meet the necessary standards for readability and understandability of health information [17].

To address the gap between the complexity of scientific evidence and the public, many organizations, including Cochrane, dedicate a lot of effort to enhancing the quality of health information available to the public [18]. Cochrane Database of Systematic Reviews is recognized as a highly reliable source for evaluating the effectiveness of health interventions [19]. Cochrane systematic reviews provide both a scientific abstract for professionals and a plain language summary (PLS) for the lay public [20]. Studies have consistently demonstrated that PLSs, which are authored by the same researchers who write the corresponding scientific abstracts, tend to exhibit readability levels that exceed those recommended for health information intended for a general audience [20-24]. In our previous study, we found that Cochrane PLSs for oncology interventions not only required a reading proficiency well above the average public level but also used language that lacked engagement, potentially reducing the reader’s interest [21]. Additionally, the PLSs frequently contained ambiguous or insufficiently clear conclusions regarding the efficacy of the interventions assessed in the systematic review [21]. This lack of clarity can leave readers, especially patients and nonspecialists, uncertain about treatment benefits or outcomes, thus diminishing the utility of these summaries in supporting informed health decisions. In addition, we have to bear in mind that the language people use plays a vital role in processing and interpreting information in text, as well as in shaping psychological responses to information and influencing whether the reader will perceive the content as more relatable [25,26]. Given the significant challenges associated with the readability and clarity of PLSs, it is important to explore innovative solutions that can enhance the communication of health information to the lay public.

Artificial intelligence (AI) tools recently emerged as a tool to help generate textual outputs relevant to health care, with the potential to revolutionize the medical sector [27,28]. A standout example of AI in action is the Chat Generative Pretrained Transformer (ChatGPT), a chatbot that operates on the Generative Pretrained Transformer technology [29]. This technology is a type of large language model (LLM) with over 175 billion parameters, capable of understanding and generating text that mimics human conversation [30]. ChatGPT has been trained on a wide variety of internet content, such as books, articles, and websites [31,32]. Its fine-tuning process includes reinforcement learning from human feedback, enhancing its ability to grasp complex user intents and respond accurately to a wide array of tasks, including those related to medical inquiries [30,33]. The deployment of natural language processing models like ChatGPT in the health care field promises to significantly improve access to medical information for both professionals and patients [29,34,35].

Recognizing the challenge posed by the low readability of PLSs authored by researchers for Cochrane systematic reviews, particularly in the context of oncology interventions, we sought to investigate the potential of using AI LLMs, specifically ChatGPT as one of the most accessible tools today, to create PLSs that are more relatable to the public. Cochrane reviews are renowned for their rigorous methodologies and comprehensive analyses; however, this complexity often results in PLSs that may be difficult for a lay audience to understand [20,21]. By using ChatGPT, we aimed to improve the readability and the linguistic characteristics of these summaries, so that they effectively communicate critical findings, methodologies, and implications in a manner that is clear and engaging for nonexpert audiences.

## Methods

### Study Design and Data Sources

In this study, we analyzed the readability, linguistic characteristics, and conclusiveness of the PLSs generated by ChatGPT from corresponding scientific abstracts of Cochrane systematic reviews of oncology interventions. We then compared them to the readability and linguistic characteristics of the original PLSs, as well as with the conclusiveness of the corresponding scientific abstracts. The dataset included 275 PLSs and corresponding scientific abstracts of Cochrane systematic reviews about oncology interventions up to February 2019 from our previous study [21]. In that study, we assessed the language characteristics of PLSs of Cochrane systematic reviews of oncology interventions in comparison with corresponding Cochrane scientific abstracts. We used this dataset as it included the scientific abstracts for which the conclusiveness of the efficacy of interventions was already assessed, so that we could compare the conclusiveness of the AI-generated PLS with that of the original scientific abstracts from which it was created. Cochrane systematic reviews included in the dataset addressed came from Cochrane review groups focused solely on oncology: Breast Cancer; Childhood Cancer; Colorectal Cancer; Gynaecological, Neurooncology, and Orphan Cancer; Haematological Malignancies; and Lung Cancer. These groups served as representatives of different clinical cancer types. Systematic reviews that did not address intervention studies were excluded. Summaries from the dataset have been analyzed in terms of their Simple Measure of Gobbledygook (SMOG) index, linguistic characteristics (word count and percentage of words related to different emotions), and the category of conclusiveness [21]. The full dataset is publicly accessible via the Open Science Framework [36].

We used the STROBE (Strengthening the Reporting of Observational Studies in Epidemiology) checklist for reporting the results of this study.

### Generation of PLSs by ChatGPT

We used a subscription chatbot, ChatGPT (version 4; Open AI) [37]. At the moment of our research, training data for ChatGPT included information up to April 2023.

**Figure 1.**
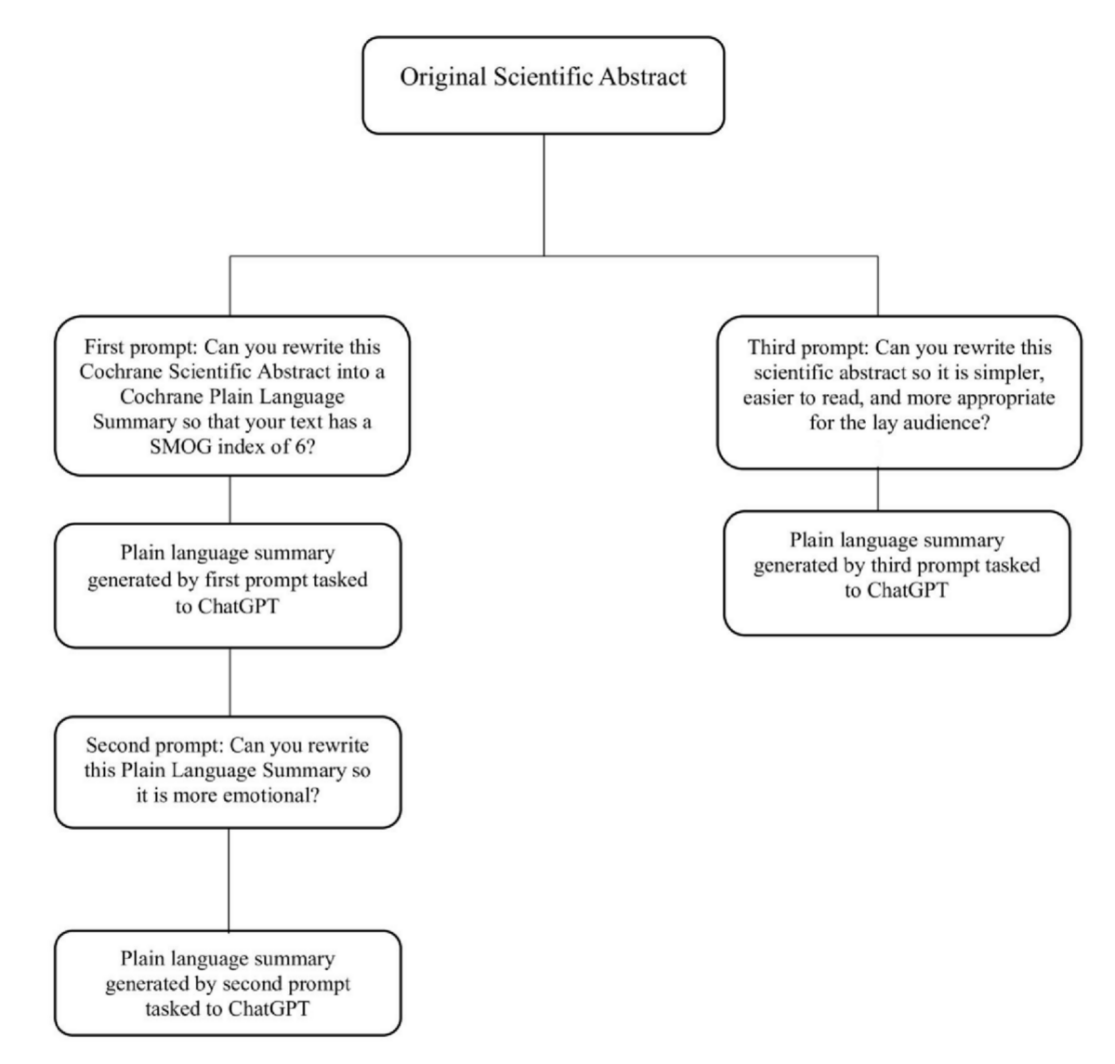
Generation of Cochrane plain language summaries by ChatGPT. SMOG: Simple Measure of Gobbledygook.

We formed 3 prompts, and an author (JŠP) asked ChatGPT each prompt for each PLS separately. Before asking these questions, we asked ChatGPT to explain what a SMOG index is. ChatGPT correctly described the readability measure, its formula, and the interpretation of results, confirming that ChatGPT was adequately familiar with the SMOG index. We then used the following prompts (Figure 1):

1. Can you rewrite this Cochrane Scientific Abstract into a Cochrane Plain Language Summary so that your text has a SMOG index of 6?
2. Can you rewrite this Plain Language Summary so it is more emotional?
3. Can you rewrite this scientific abstract so it is simpler, easier to read, and more appropriate for the lay audience?

In the first prompt, we asked ChatGPT to rewrite the scientific abstract into PLS with a SMOG index of 6, because the official recommendation of the American Medical Association and National Health Institute is that the health information should be written at the reading level of the sixth grade in the US education system [16,38].

For the second prompt, we continued the conversation with ChatGPT that started in the first prompt. Since we found in our previous study that PLSs had language that was more emotional compared with the corresponding scientific abstracts and that PLSs that have a higher percentage of emotional tone have better readability [21], we used the PLS that was provided to ChatGPT in first prompt and asked ChatGPT to rewrite it again so that is more emotional, so this prompt was included to assess if adding emotional resonance could enhance reader engagement and relatability.

In prompt 3, similar to prompt 1, we asked ChatGPT to simplify the scientific abstract, but without defining the SMOG index. This prompt was designed to explore if using simpler language alone (without a specified readability index) would yield results with improved accessibility while retaining the essential information and nuance necessary for an accurate understanding of oncology topics. When tasking this prompt, we started a new chat with ChatGPT, so that it did not rely on the previous answer.

After that, for each PLS provided by ChatGPT, we measured its readability, expressed as SMOG index, and the following linguistic characteristics: word count and percentage of words related to authenticity, clout, emotional, and analytic tone. We analyzed only the first answers generated by ChatGPT for each of the 3 prompts and did not ask ChatGPT to revise the texts again. To be sure that the content of the PLS provided the same facts as the corresponding scientific abstract, we also checked the conclusiveness of each generated PLS, that is, checked whether there was any difference with the original conclusiveness category for the scientific abstract, determined in our previous study [21]. Multimedia Appendix 1 contains a supplementary table with examples of prompts.

### SMOG Index

The readability of summary formats in English was assessed using the SMOG index [39]. SMOG index assesses the readability of certain content by counting polysyllabic words and the result is presented as the number of years of education required to understand a given text [39]. It is considered to be suitable for health information due to its consistent results, higher expected comprehension levels, application of recent validation criteria for estimating reading grade levels, and ease of use [39]. SMOG index for PLSs in English was calculated using a web-based tool “WebFX Readability Test Tool” [40]. The tool’s reliability and accessibility made it a suitable choice for evaluating the readability of PLSs in our study. Regarding SMOG index interpretation, the official recommendation of the American Medical Association and National Health Institute is that health information should be written at the reading level of the sixth grade in the US education system [16,38].

### Linguistic Characteristics

PLSs generated by ChatGPT were analyzed regarding their linguistic characteristics, using the Linguistic Inquiry and Word Count (LIWC) [41], a software tool designed to analyze a given text by comparing each word to its predefined dictionary. The tool categorizes text words into 4 main variables: analytical, clout, authenticity, and emotionality shared in the tone of the text, expressed as the percentage of words from the text in a particular category. The analytical thinking category is based on recognizing words associated with logic or connecting concepts and putting them into a relationship. Greater use of words related to analytical thinking is related to cognitive complexity and abstract thinking [26]. Clout speech is a variable that refers to the use of terms that denote self-confidence, leadership, or social status. A higher proportion of such words suggests that the author speaks from a position of expertise and certainty in what is stated, and a lower proportion suggests a style of presenting information that is humbler [42]. Authenticity is determined by the percentage of words related to personality, such as the use of personal nouns in the first person (“I”, “my”, and “mine”), present tense, and relative adverbs (near, now). The use of these words is connected to writing that is more personal and honest [43]. Emotional share relates to how positive the tone is according to the words used. A score of 100 in emotional tone would mean the tone is maximally positive, while a score of 50 means an even balance of positive and negative emotion words [44].

### Conclusiveness

The category of conclusiveness for each ChatGPT-generated PLS was analyzed by JŠP and checked by IB. Data extraction spreadsheet was tested by 2 authors (JŠP and IB). One author extracted the data, and the other one independently reviewed the data in a 10% random sample of PLSs and corresponding scientific abstracts. Then, it was checked whether the entry in the table was correct. Interobserver agreement was high (κ range 0.80 to 1.00, 95% CI 0.84‐1.00). We resolved the differences in rating through the discussion with a third author (AM) before full data extraction.

The conclusiveness of statements about efficacy and safety was categorized into 3 categories [45]:

1. Conclusive: positive conclusive (there was moderate- or high-quality evidence indicating the effectiveness or safety of the intervention; ie, the drug was proven effective/safe); negative conclusive (there was moderate- or high-quality evidence indicating that the intervention is ineffective or harmful, or authors advised against the intervention/comparison or it is not recommended); or equal conclusive (the interventions analyzed were equally effective and safe).
2. Inconclusive: positive inconclusive (there was evidence suggesting effectiveness or safety, but it is of low quality or inconclusive, and the authors suggest that more research is needed); negative inconclusive (there was evidence of ineffectiveness or harm (evidence demonstrating that there was no effect or that the intervention was not safe) or authors urged against the intervention or comparison, or it is not recommended; however, the evidence is of low quality or inconclusive, or authors state that more research is needed); or equal inconclusive (the interventions appeared to be similarly effective and safe, but the evidence was of lower quality or inconclusive, and the authors suggest that more research is needed).
3. Unclear: no evidence (there was no evidence as the search did not retrieve any randomized controlled trials, ie, empty reviews); no opinion (the authors did not offer any opinion or judgment); and unclear (the authors did not give a clear conclusion or state that the more research is required).

Based on these criteria, we defined a category of conclusiveness for each of the derived PLS, and then the category of conclusiveness was compared with those from the original scientific abstracts to check whether they match and give the same conclusion about the effectiveness or safety of the intervention [21].

### Statistical Analysis

#### Descriptive Statistics

The data on readability, word count, and linguistic characteristics were assessed as numeric variables. As the data deviated from normal distribution, the results were presented as medians and 95% CI and were presented on original PLS and across 3 prompt groups. The data on conclusiveness was assessed as frequencies and was presented on a bar chart across 3 different prompt groups.

#### Group Comparison

The results from the analysis of ChatGPT-generated PLSs were compared with the already published data for the original PLSs and scientific abstracts [21]. Since all versions were derived from the same PLS, the results were treated as within the subjects’ group under different conditions. Since the within-subjects ANOVA was not appropriate due to the deviations in the normality of data distribution, the comparison between groups was made using the Friedman nonparametric test for repeated measures, as nonparametric alternative and post hoc testing was made using the Conover post hoc test, since it is one of the recommended methods.

#### Statistical Software

Analyses were made using JASP (v.0.18.1.0; Jasp Team 2023) and R (v4.3.3; R core team, 2024) [46].

### Ethical Considerations

The authors did not require ethical approval as the study is based solely on publicly available summaries of Cochrane systematic reviews. The research does not involve human participants or the use of animals. This is in accordance with the ethical code of the University of Split School of Medicine (April 2009).

## Results

### Overview

We generated a total of 275 PLSs for each of the 3 ChatGPT prompts. On average, all of them had statistically fewer words than the original PLSs (Table 1).

**Table 1.** Comparison of linguistic characteristics (median, 95% CI) between different plain language summary (PLS) groups.

Variable	Original^a^, median (95% CI)	ChatGPT prompt, median (95% CI)			*P* value^b^
		First prompt: write from scientific abstract at SMOG^c^ index 6	Second prompt: make PLS from first prompt more emotional	Third prompt: write simpler PLS from original PLS	
Word count	364 (339‐388)	240 (232‐248)	285 (278‐292)	273 (266‐278)	<.001
SMOG index^d^	13.1 (12.9‐13.4)	8.2 (8.0‐8.4)	11.4 (11.1‐12)	8.7 (8.4‐8.8)	<.001
Linguistic characteristics^e^					
Analytical tone	95.5 (95.0‐95.8)	55.9 (53.6‐57.9)^f^	85.7 (84.0‐86.7)	60.9 (57.7‐63.1)	<.001
Clout	50.0 (47.7‐51.8)	67.2 (64.9‐70.9)^f^	80.3 (77.8‐83.6)	70.5 (68.0‐72.8)	<.001
Authenticity	28.6 (26.2‐31.3)	50.5 (47.0‐53.5)^f^	38.0 (34.8‐40.1)	49.4 (45.8‐54.2)	<.001
Emotional tone	22.1 (18.5‐26.2)	54.8 (51.3‐58.7)^f^^,g^	63.9 (58.6‐69.6)^f^	54.4 (51.9‐56.4)	<.001

a The results for the original PLSs are from the previous study [21].

b Friedman nonparametric test. All post hoc differences were statistically significant except those labeled with symbols in superscript.

c SMOG: Simple Measure of Gobbledygook.

d Readability was measured as a SMOG index [39]. Higher scores indicated lower readability.

e Linguistic characteristics of the text were measured using dictionary-based text word categorizations by the Linguistic Inquiry and Word Count (LIWC) [26]. The variables are presented as the percentage of words from the text in a particular category.

f Not different from prompt 3.

g Not different from prompt 2.

### SMOG Index and Linguistic Characteristics

PLSs generated by the first prompt (write at SMOG index 6) were the easiest to read, with the median SMOG index of 8.2 (95% CI 8‐8.4), and the shortest. Regarding linguistic characteristics, these PLSs had less analytical tone and more authenticity, clout, and emotional tone when compared with the original PLSs written by authors (Table 1).

PLSs generated by the second prompt (make prompt 1 more emotional), had a median SMOG index of 11.4 (95% CI 11.1‐12). Those PLSs also had more analytical tone and clout compared with the PLSs generated by the first prompt, but no difference in the emotional tone. They also used fewer words related to authenticity than the PLSs generated by the first prompt (Table 1).

PLSs generated by the third prompt (write simpler PLS from original) had a median SMOG index of 8.7 (95% CI 8.4‐8.8). Linguistic characteristics did not differ from the PLSs generated by the first prompt, but they had a less analytical and more authentic tone than the PLSs generated by the second prompt (Table 1).

Across the 3 GPT prompts, the results were consistent, without major outliers.

### Conclusiveness

**Figure 2.**
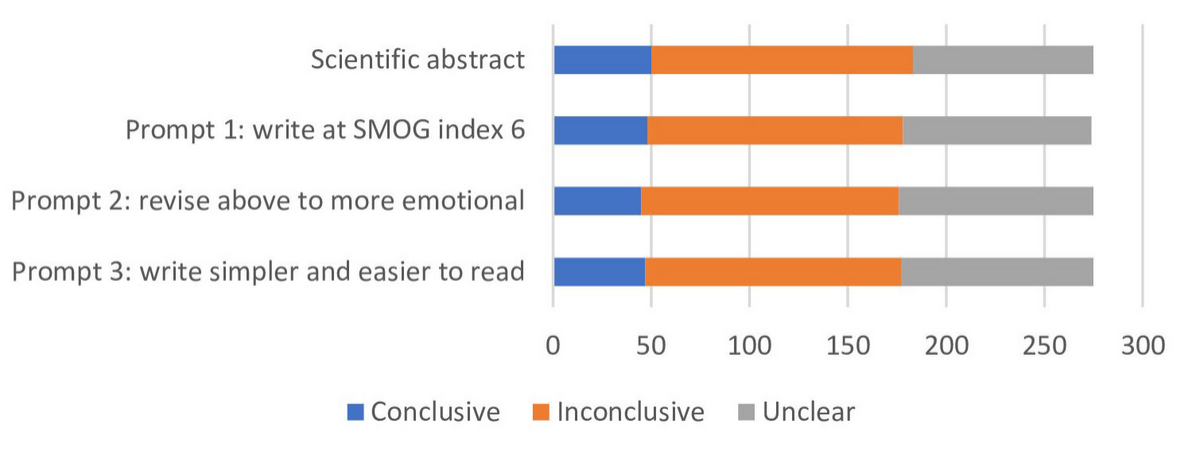
Distribution of plain language summaries according to the conclusiveness of the efficiency of interventions described in the systematic reviews across 4 different groups of writing prompts. SMOG: Simple Measure of Gobbledygook.

The category of conclusiveness of all 3 ChatGPT-generated PLSs did not differ from that of the original scientific abstract (Figure 2).

## Discussion

### Principal Findings

Our study investigated the potential of using an LLM-ChatGPT, to generate more readable and engaging PLSs for Cochrane systematic reviews in oncology. The results demonstrate that ChatGPT-generated PLSs were shorter and easier to read, as illustrated by the SMOG indices across different prompts. Across all prompts, ChatGPT-generated PLSs exhibited a decrease in analytical tone while showing higher levels of authenticity, clout, and emotional tone compared with the original PLSs. Notably, the categorization of conclusiveness in the original abstracts remained consistent in the ChatGPT-generated PLSs.

Specifically, PLSs created by the first prompt (targeting a SMOG index of 6) achieved the lowest SMOG index and the highest readability among the 3 approaches we used. The median SMOG index of the PLSs generated through prompt 1, while higher than the targeted level of 6, was still significantly lower than that of the original PLSs authored by researchers (median SMOG index 13.1).

The second prompt, which aimed to make the summaries contain text with more emotionally positive content, resulted in an increase in SMOG index and a notable shift toward analytical and clout tones, indicating that the addition of emotional language might inadvertently increase linguistic complexity. This observation is crucial because previous studies have shown that emotional resonance can improve reader engagement and comprehension [21], yet it must be balanced with readability to avoid overcomplicating the content.

The third prompt again managed to improve the readability of the summaries. Despite differences in readability and tone, the conclusiveness about the efficacy of the intervention in ChatGPT-generated PLSs remained consistent with that of the original scientific abstracts. This consistency is encouraging, as it suggests that ChatGPT can maintain the integrity of the original scientific conclusions while rephrasing content for a lay audience.

PLSs generated by ChatGPT had language characteristics more suitable for the lay population than the original PLSs from the published Cochrane systematic reviews [21]. They had fewer words and better readability—from the median of 8.2 to 11.2 compared with the original 13.1 SMOG index, bringing it closer to the recommended sixth-grade reading level for the health information intended for the lay public [16]. However, it is not clear whether we can expect at all for PLSs from oncology to be at the recommended reading level, as it is not sometimes possible to replace complex scientific expressions or names of the drugs (eg, trastuzumab-deruxtecan) without altering the meaning and jeopardizing the translation of the information to the reader in the correct way.

Regarding the linguistic content of the PLSs [16,42-44], those generated by ChatGPT had lower content with an analytical tone, meaning that they did not relate so much to abstract thinking or cognitive complexity as the original PLSs. They also had a higher positive emotional tone (a score over 50 for all ChatGPT-generated PLSs) than the original PLSs, which had a predominantly negative emotional tone (median of 22 out of maximum 100). ChatGPT-generated PLSs also had higher clout, meaning that the information came from the position of expertise and certainty, and they used more personal nouns, present tense, and relative adverbs, increasing authenticity tone, that is, making it more personal and honest. In this way, ChatGPT-generated PLSs could influence the subjective experience of people and their engagement in the given text when they process the information in the PLSs with respect to their opinion about the truthfulness of the information or their confidence in the information [25]. These cognitive processes are very important in reacting to health information, as it has been shown that patients adhere more to advice from doctors that contains more positive emotions [47].

### Comparison to Prior Studies

ChatGPT-generated PLSs were closer in their characteristics to the press releases of Cochrane systematic reviews, written by professional writers. A study of Cochrane systematic reviews that had an official press release showed that these press releases were written in a more conversational and emotional language than the scientific abstracts or PLSs in different languages, making them more engaging [22]. ChatGPT-generated PLSs had similar qualities, without losing the conclusiveness of the message, making them more suitable for health evidence translation to the patients and the general public.

### Strength and Limitations

Regarding the strengths of the study, this study is among the first to evaluate the use of AI for generating PLSs in oncology, focusing on readability, linguistic characteristics, and consistency with original scientific conclusions. The use of multiple prompts provided a nuanced understanding of how prompt design influences AI-generated content.

These results must be considered in light of several limitations. First, we used PLSs from a single source, the Cochrane Library, and these PLSs were written by different authors. However, summaries from the Cochrane Library have the same format of presenting health information and specific guidance for writing PLSs [48], making them comparable. Second, we analyzed the PLSs in English only since it was the only common language for the summaries in oncology systematic reviews. The focus on English ensured uniformity in linguistic analysis, avoiding inconsistencies in translation processes. Third, a notable limitation of this study is that it required ChatGPT to generate PLSs from scientific abstracts and not from the complete texts of Cochrane systematic reviews. Typically, PLSs are derived from the full content of the reviews [48], which provides a more thorough understanding of the study’s findings, methodologies, and contextual factors. Reliance only on scientific abstracts may lead to PLSs lacking depth and detail. Fourth, ChatGPT-4 was developed using a diverse dataset of publicly available information spanning multiple domains and at the time of our study, its information is limited to publicly available sources up to 2021 [49]. OpenAI does not specify the exact content of medical information included or the precise time frame of the dataset [49]. We used ChatGPT-4, the subscription version (unlike its predecessor ChatGPT-3.5), the most advanced and widely available version of ChatGPT during the study period, ensuring access to its latest capabilities, but it is available only to people willing to pay a monthly subscription. For the second prompt in our study, we relied on the answers from ChatGPT provided in the first prompt. For all 3 prompts, we did not ask the system to further rephrase the text but analyzed only the first output. Different AI models vary in training, algorithms, and capabilities, making the use and results of one model not universally applicable to others [50]. We did not use other AI tools such as Microsoft Bing AI, Bard, Jasper, or ChatSonic, which could have given different results. Microsoft Bing AI can only process up to 2000 characters [51], which is not suitable for summaries. Bard was not available in Croatia at the time of conducting the study [52], and Jasper and ChatSonic offered only paid subscriptions for which we did not have sufficient resources [53,54]. Additionally, we did not use specialized AI tools designed specifically for creating PLS, such as Sorcero’s solution [55] or Putnam Associates’ generative AI approach for PLSs [56]. Our decision not to use these specialized tools stemmed from a focus on general AI models that are more accessible to a wider range of users and our intent to evaluate the performance of widely available, nonspecialized AI for PLS generation. Fifth, it has to be kept in mind that, while our study assessed linguistic characteristics such as clout, analytical tone, authenticity, and emotional tone, it is equally important to consider the cultural and emotional sensitivities of the target audience [57]. AI models like ChatGPT-4 are trained on extensive datasets that may not fully capture the nuances of various cultural backgrounds. Consequently, the generated PLSs might lack the cultural relevance or sensitivity necessary to effectively communicate with all segments of the lay public. Further research should include PLSs from multiple sources to assess the generalizability of AI-generated PLSs across diverse formats and writing styles and explore the potential of AI tools in generating PLSs in languages other than English, to support Cochrane efforts to provide health information in 20 different languages [58].

### Future Directions

What conclusions and recommendations can be drawn from our study? Our study is one of the first tests of AI language tools in creating health information from complex health evidence synthesis that is suitable for the lay public. The real-world implementation of medical AI interventions generally lacks high-quality evidence, as a recent systematic review identified 65 randomized controlled trials evaluating AI interventions, but only 7 with chatbots as an intervention [59]. We do not think that, at the current level of development, ChatGPT can replace evidence synthesis in real time, as it does not get updates in real time [60] and there is a potential for bias in the training data, which can result in biased or inaccurate responses [61]. In addition, text generated from the training data can have several other issues besides bias, such as plagiarism [62], lack of context, as well as underestimation of novelties in medicine that are important but may be less represented in web sources [63].

On the other hand, ChatGPT and other generative AI tools may be useful in ensuring the quality and appropriateness of the summary information for health evidence synthesis, such as Cochrane systematic reviews. Although Cochrane has clear guidance on writing PLSs [48], the evidence shows that they are not adequately implemented and published PLSs are not at the desired level of clarity and quality [64]. It seems that the authors of Cochrane systematic reviews have difficulties in translating their results into a language that is suitable for the lay public. This affects not only the usefulness of the PLSs but also their translations into a number of languages, where it is not clear whether there is a further loss to the clarity and understandability of the message to the lay public [22]. Readability metrics like the SMOG index provide an indication of text complexity but do not guarantee comprehension by the intended audience. This gap highlights the need for future research to evaluate the effectiveness of AI-generated PLSs in real-world settings and to determine their actual understandability among diverse patient populations.

### Conclusions

Having all that in mind, ChatGPT may be a valuable tool in helping create content designed for the lay public. Cochrane should further explore the use of ChatGPT in generating PLSs, either as a tool for the authors or as an independent, systemic tool to generate high-quality, high-fidelity PLSs, also ensuring that the main message of health information is unchanged and accurate.

## Supplementary material

10.2196/63347 Multimedia Appendix 1 Example sheet.

10.2196/63347 Checklist 1 STROBE (Strengthening the Reporting of Observational Studies in Epidemiology) checklist.

## Abbreviations:

AI: artificial intelligence
LIWC: Linguistic Inquiry and Word Count
LLM: large language model
PLS: plain language summary
SMOG: Simple Measure of Gobbledygook
STOBE: Strengthening the Reporting of Observational Studies in Epidemiology
